# TMEM165 a new player in proteoglycan synthesis: loss of TMEM165 impairs elongation of chondroitin- and heparan-sulfate glycosaminoglycan chains of proteoglycans and triggers early chondrocyte differentiation and hypertrophy

**DOI:** 10.1038/s41419-021-04458-1

**Published:** 2021-12-20

**Authors:** Sajida Khan, Malak Sbeity, François Foulquier, Lydia Barré, Mohamed Ouzzine

**Affiliations:** 1 UMR7365 CNRS-University of Lorraine, Biopôle, Faculty of Medicine, Vandoeuvre-lès-Nancy, Nancy, France; 2 UMR8576 CNRS-University of Lille, Lille, France

**Keywords:** Glycobiology, Diseases

## Abstract

TMEM165 deficiency leads to skeletal disorder characterized by major skeletal dysplasia and pronounced dwarfism. However, the molecular mechanisms involved have not been fully understood. Here, we uncover that TMEM165 deficiency impairs the synthesis of proteoglycans by producing a blockage in the elongation of chondroitin-and heparan-sulfate glycosaminoglycan chains leading to the synthesis of proteoglycans with shorter glycosaminoglycan chains. We demonstrated that the blockage in elongation of glycosaminoglycan chains is not due to defect in the Golgi elongating enzymes but rather to availability of the co-factor Mn^2+^. Supplementation of cell with Mn^2+^ rescue the elongation process, confirming a role of TMEM165 in Mn^2+^ Golgi homeostasis. Additionally, we showed that TMEM165 deficiency functionally impairs TGFβ and BMP signaling pathways in chondrocytes and in fibroblast cells of TMEM165 deficient patients. Finally, we found that loss of TMEM165 impairs chondrogenic differentiation by accelerating the timing of Ihh expression and promoting early chondrocyte maturation and hypertrophy. Collectively, our results indicate that TMEM165 plays an important role in proteoglycan synthesis and underline the critical role of glycosaminoglycan chains structure in the regulation of chondrogenesis. Our data also suggest that Mn^2+^ supplementation may be a promising therapeutic strategy in the treatment of TMEM165 deficient patients.

## Introduction

Glycosylation is one of the most common and important posttranslational modifications of proteins [1, 2]. Genetic defects in protein glycosylation can lead to Congenital Disorders of Glycosylation (CDGs) which is a group of inherited diseases associated with a broad variety of pathological symptoms [3]. Among these, the recently identified CDG subtype linked to mutations in *TMEM165* (transmembrane protein 165) [4]. Mutation of the yeast ortholog of *TMEM165*, named *Gdt1* induced sensitivity to high Ca^2+^external concentrations, suggesting its participation to Ca^2+^ transport and reduction of the concentration of Ca^2+^ in the cytosol [3]. *TMEM165* gene deficiency was associated with a slight defect in sialylation and galactosylation of *N*-glycans in TMEM165-deficient patients. Although, the transport activity of TMEM165 in human cells have not been demonstrated yet, several indirect observations suggest that TMEM165 may be involved in maintaining Golgi Ca^2+^, H^+^, Mn^2+^ homeostasis [5].

Different mutations were detected in *TMEM165* in the patients suffering from CDGs, including missense mutations R126H and R126C located in the putative lysosomal targeting motif ^124^YNRL^127^, the mutation E108G lying in the highly conserved motif ^108^ELGDKT^113^, the double mutations R126C/G304R and intronic splice mutation leading to skipping of the exon four and generation of truncated protein. The major clinical findings in the individuals with a homozygous splice mutation leading to total loss of TMEM165 protein are severe psychomotor retardation, major skeletal dysplasia, and pronounced dwarfism. Interestingly, similar phenotype was observed in patients harboring genetic mutations in genes encoding enzymes involved in the synthesis of proteoglycans (PGs) [6–9]. Here, we generated *tmem*165-knockout pre-chondrocyte mouse ATDC5 and human HEK293 cells and showed that knockdown of TMEM165 resulted in profound deficiency in polymerization of heparan-sulfate (HS) and chondroitin-sulfate (CS) glycosaminoglycan (GAG) chains of PGs. We demonstrated that defects in GAG elongation was rescued by supplementation of culture cell medium with the divalent cation Mn^2+^, suggesting a role of TMEM165 in the homeostasis of Golgi Mn^2+^. Importantly, we found that loss of TMEM165 functionally impairs TGFβ/BMP signaling pathways and accelerates timing of Ihh expression leading to early differentiation and hypertrophy of chondrocytes.

## Results

### Loss of TMEM165 impairs elongation of heparan- and chondroitin-sulfate chains

**Fig. 1:**
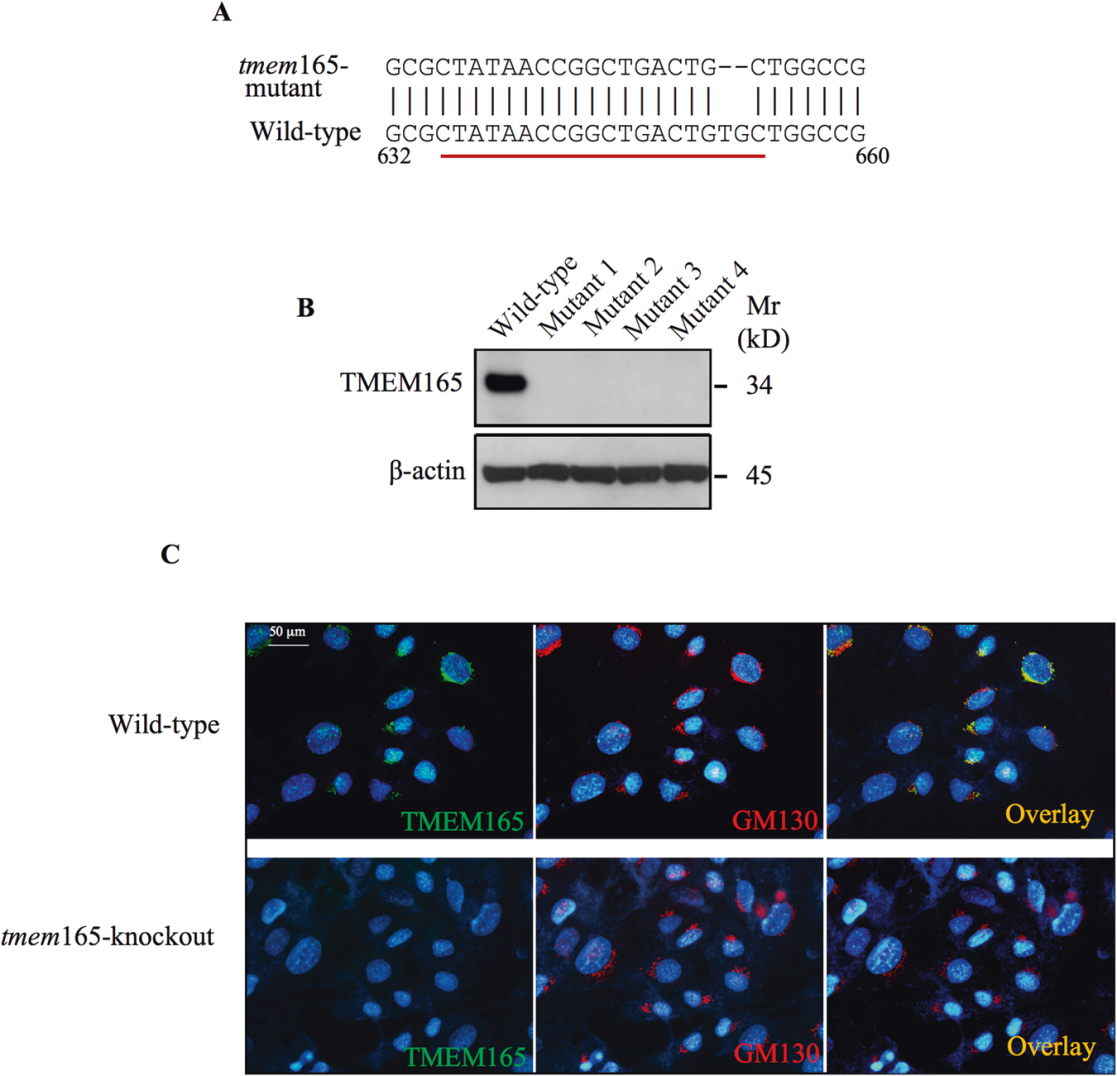
CRISPR/Cas9 knockdown of TMEM165. **A** Alignment of TMEM165 targeted sequence from wild-type and mutant mouse ATDC5 cells. **B** Detection of TMEM165 in cell lysates from wild-type and *tmem*165-knockout mouse ATDC5 cells (mutant 1, 2, 3 and 4) using anti-TMEM165 specific antibodies. β-actin was used as loading control (*n* = 3). **C** Immunofluorescence analysis of the expression of TMEM165 in mouse ATDC5 control and *tmem*165-knockout cells using antibodies against TMEM165 (green). GM130 (red) was used as a Golgi marker. The nucleus was stained with DAPI (blue) (*n* = 3). Digital images were captured with an inverted microscope, Leica DMI3000. Representative images from three independent experiments are shown. Scale bar: 50 μm.

Genetic mutations or knockout of glycosyltransferases involved in GAG synthetic pathway cause chondrodysplasia. To determine the link, if any, between TMEM165 deficiency and the synthesis of PGs, we generated *tmem*165-knockout prechondrogenic mouse ATDC5 cell line using CRISPR-Cas9 technique. *tmem*165-knockout clones harboring deletion mutations were selected for further studies (Fig. 1A). As expected, expression of a polypeptide of about 35 kDa corresponding to TMEM165 was detected in wild-type mouse ATDC5 cells, whereas no polypeptide was revealed by the anti-TMEM165 antibodies in *tmem*165-knockout cells (Fig. 1B). To further confirm these results, immunofluorescence analysis of the expression of TMEM165 was carried out in wild-type and in *tmem*165-knockout mouse ATDC5 cells. As shown in Fig. 1C TMEM165 is clearly detected in wild-type mouse ATDC5 cells displaying a perinuclear Golgi distribution and colocalizes with the Golgi (GM130) marker. However, no staining with anti-TMEM165 was observed in *tmem*165-knockout mouse ATDC5 cells, whereas the Golgi marker GM130 was clearly detected (Fig. 1C), indicating that the expression of TMEM165 is knocked down in these cells.

**Fig. 2:**
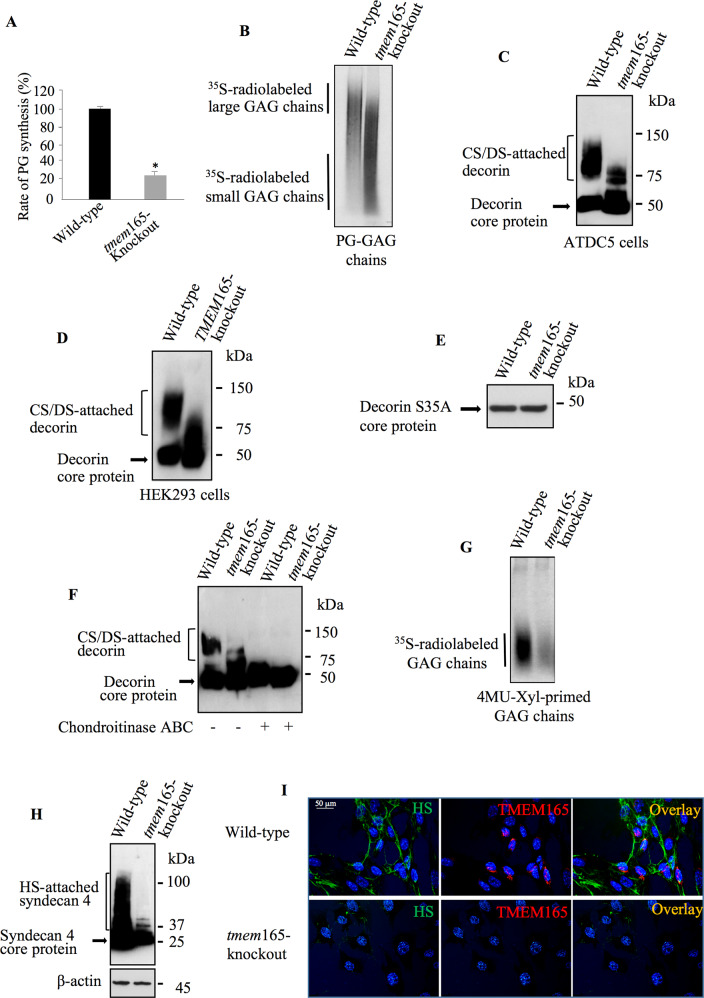
GAG chain elongation is impaired in TMEM165-deficient cells. **A** PG anabolism evaluation in wild-type and *tmem*165-knockout mouse ATDC5 cells by measurement of the incorporation rate of [^35^S]-sulfate into the GAG chains (*n* = 3). **B** SDS-PAGE and autoradiography analysis of neosynthesized radiolabelled PG-GAG chains in wild-type and *tmem*165-knockout mouse ATDC5 cells (*n* = 3). **C** Detection of decorin in conditioned medium of wild-type and *tmem*165-knockout mouse ATDC5 cells and (**D**) in wild-type and *TMEM*165-knockout HEK293 cells (*n* = 3). **E** Detection of decorin S34A mutant lacking GAG chain in conditioned medium of wild-type and mutant mouse ATDC5 cells (*n* = 3). **F** Analysis of the sensitivity to degradation by chondroitinase ABC of GAG chains of decorin in conditioned medium of wild-type and *tmem165*-knockout mouse ATDC5 cells (*n* = 3). **G** SDS-PAGE and autoradiography analysis of neosynthesized radiolabelled GAG chains primed with 4MU-Xyl in wild-type and *tmem*165-knockout mouse ATDC5 cells (*n* = 3). **H** Detection of HA-syndecan 4 in cell lysates of wild-type and *tmem*165-knockout mouse ATDC5 cells transfected with HA-syndecan-4 expression vector. β-actin was used as loading control. (*n* = 3) **I** Immunofluorescence analysis of cell surface HS GAG chains using anti-HS specific antibodies (green) and of the expression of TMEM165 (red) in wild-type and *tmem*165-knockout mouse ATDC5 cells. The nucleus was stained with DAPI (blue). Digital images were captured with an inverted microscope, Leica DM13000. Representative images from three independent experiments are shown. Scale bar: 50 μm.

To uncover the link, if any, between TMEM165 and PGs, we evaluated the level of PG synthesis in wild-type and *tmem*165-knockout mouse ATDC5 cells by using metabolic incorporation of radiolabelled [^35^S]-sulfate into GAG chains. The results showed a decrease of about 70% in the rate of PG synthesis in *tmem*165-knockout cells, compared to wild-type cells (Fig. 2A). Interestingly, SDS-PAGE analysis of radiolabelled PG-GAG chains showed predominance of shorter GAG chains in *tmem*-knockout cells, compared to wild-type (Fig. 2B). To determine whether the defects in GAG synthesis affects both CS- and HS-GAG chains, we used decorin and syndecan 4 as reporter for the synthesis of CS-PGs and HS-PGs, respectively. Transfection of wild-type and *tmem*165-knockout mouse ATDC5 cells with decorin expression vector resulted in the secretion of CS/DS-attached decorin in the culture medium as shown by Western blot (Fig. 2C). However, there is a loss of higher sized CS/DS-attached decorin species (80–150 kDa) in *tmem*165-knockout mouse ATDC5 cells as evidenced by predominance of decorin with smaller size GAG chains (70 to 80 kDa) (Fig. 2C). Similar results were obtained using *TMEM165*-knockout HEK293 cells (Fig. 2D). This indicates that defects in PG synthesis produced by the loss of TMEM165 is not cell type specific. To rule out an effect of TMEM165 on decorin core protein expression or secretion, we generated and expressed a decorin mutant lacking GAG-attachement site by mutation of serine residue at position 34, which is used for the attachment of the CS-GAG chain on the core protein, to alanine residue (S34A). Expression of the mutant decorin S34A in wild-type mouse ATDC5 cells led, as expected, to the secretion of a polypeptide of about 50 kDa corresponding to decorin core protein without attached GAG chain (Fig. 2E). Of note, *tmem*165-knockout mouse ATDC5 cells secreted mutant decorin at similar amount to that produced by wild-type cells. These data indicate that TMEM165 deficiency did not affect the synthesis of decorin core protein. Altogether, these results demonstrate that the loss of TMEM165 affects the synthesis of GAG chain attached to decorin core protein. Noteworthy, treatment of decorin, produced in *tmem*165-knockout cells, with chondroitinase ABC led to change in the migration pattern in SDS-PAGE from a smear to a single band corresponding to decorin core protein (Fig. 2F), indicating that decorin from mutant cells is sensitive to chondroitinase ABC and therefore contains CS-GAG chains, but shorter in size. To confirm that GAG chain synthesis is affected, we used 4-Methylumbelliferyl-β-D-xylopyranoside (4MU-Xyl) as acceptor substrate for the synthesis of GAG chains in the presence of [^35^S]-sulfate to metabolically radiolabel the newly synthesized GAG chains. As shown in Fig. 2G, wild-type mouse ATDC5 cells produced significant amount of 4MU-Xyl primed radiolabelled GAG chains, whereas *tmem*165-knockout mouse ATDC5 cells produced only few amounts, indicating that the synthesis of GAG chains is impaired in *tmem*165-knockout cells. We next investigated whether the synthesis of HS-GAG chains is also altered in *tmem*165-knockout cells. For this purpose, we expressed syndecan 4, a cell surface HSPG, in *tmem*165-knockout and wild-type mouse ATDC5 cells. As shown by Western blot, syndecan 4 expressed in wild-type mouse ATDC5 cells exhibited a smear pattern in SDS-PAGE corresponding to HS-attached syndecan 4, whereas in tmem165-knockout cells the smear was strongly reduced in size, compared to wild-type cells indicating a loss of higher sized HS-attached syndecan 4 species in *tmem*165-knockout mouse ATDC5 cells (Fig. 2H). These results indicate that elongation of HS chains is impaired in mutant cells. To further confirm this result, we carried out indirect immunofluorescence analysis of cell surface HSPGs in wild-type and *tmem*165-knockout cells, using anti-HS monoclonal antibody 10E4. Prominent staining of the cell membrane was observed in wild-type cells (green), whereas very low signal could be observed in *tmem*165-knockout cells (Fig. 2I). When cells were probed with anti-TMEM165 antibodies, efficient expression of the protein was observed in wild-type cells (red), whereas no staining was detected in *tmem*165-knockout cells (Fig. 2I). Altogether, these results revealed a key role of TMEM165 in the synthesis of both HS- and CS-GAG chains of PGs.

### Overexpression of polymerizing enzymes did not overcome elongation defects produced by loss of TMEM165

**Fig. 3:**
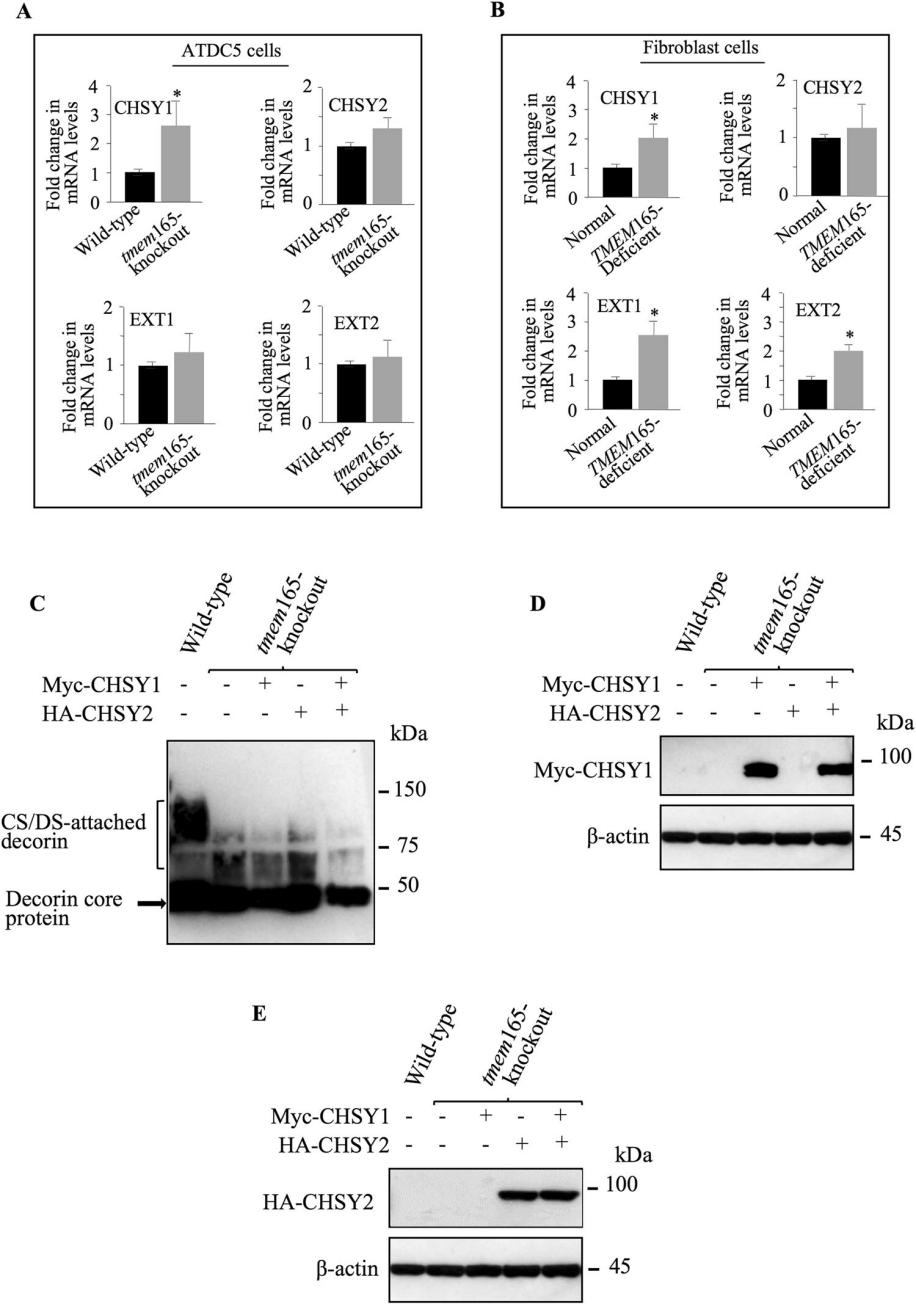
Expression of CS polymerizing enzymes did not overcome GAG elongation defects in *TMEM*165-deficient cells. **A** Fold changes in mRNA expression of CS polymerizing enzymes CHSY1 and CHSY2, and HS polymerizing enzymes EXT1 and EXT2 in *tmem*165-knockout mouse ATDC5 cells normalized to wild-type mouse ATDC5 cells and **B** in *TMEM*165-deficient fibroblasts normalized to fibroblast control cells. qPCR values were normalized for the housekeeping gene ribosomal protein S29 and are expressed as the relative expression compared with control. Data are expressed as mean ± S.D. Statistical analysis was performed with an unpaired Student’s *t* test (*n* = 3; *p < 0.05; **p < 0.01). **C** Detection of decorin in conditioned medium of wild-type and mutant ATDC5 cells transfected with empty vector, Myc-tagged CHSY1, HA-tagged CHSY2 or Myc-tagged CHSY1 and HA-tagged CHSY2 along with decorin expression vector. **D** Detection of CHSY1 and (**E**) of CHSY2 in wild-type and *tmem*165-knockout mouse ATDC5 cells. β-actin was used as loading control (*n* = 3). Representative images from three independent experiments are shown.

Given that CS and HS synthetic pathways involve several enzymes each of which is involved in a specific step of the synthesis process, we sought to determine whether impaired elongation of CS- and HS-GAG chains in *tmem*165-knockout cells resulted from defects in the gene expression of CS and HS polymerizing enzymes. To this end, RT-qPCR analysis were performed using mRNA from *tmem*165-knockout and wild-type mouse ATDC5 cells to evaluate gene expression of CS polymerizing enzymes, CHSY1 and CHSY2 and of HS polymerizing enzymes, EXT1 and EXT2. The results showed that mRNA levels of the enzymes are similar or higher in *tmem*165-knockout mouse ATDC5 cells compared to wild-type (Fig. 3A). Similar results were observed in TMEM165-deficient patient fibroblasts, compared to normal fibroblast cells (Fig. 3B), suggesting that impaired elongation of CS- and HS-GAG chains in mutant cells is not due to downregulation of gene expression of the polymerizing enzymes. However, we cannot rule out a decrease in the protein level that may result from increased protein instability and/or degradation. Given that the level of protein expression of glycosyltransferases involved in GAG synthetic pathway is very low and their detection by antibodies (commercially available) is very challenging, it is, therefore, difficult to evaluate and compare the protein levels of CS and HS polymerizing enzymes in normal and mutant cells. We can however hypothesize that overexpression of these enzymes may overcome the instability or degradation of these proteins, if any. We then designed expression vectors for Myc-tagged CHSY1 and HA-tagged CHSY2 and used them to overexpress each of the two CS elongation enzymes individually or together in *tmem*165-knockout mouse ATDC5 cells. As shown in Fig. 3C, *tmem*165-knockout cells transfected with CHSY1, CHSY2 or CHSY1 and CHSY2 expression vectors produced decorin with reduced size, compared to that produced in wild-type cells. The expression of CHSY1 and CHSY2 was confirmed by Western blot in all the cells transfected (Fig. 3D, E). These data indicated that overexpression of CHSY1 and CHSY2 individually or together did not rescue CS elongation in *tmem*165-deficient cells, suggesting that defects in CS polymerization process did not result from reduced protein expression of the polymerizing enzymes.

### Manganese rescues the synthesis of chondroitin-sulfate and heparan-sulfate GAG chains in TMEM165-deficient cells

**Fig. 4:**
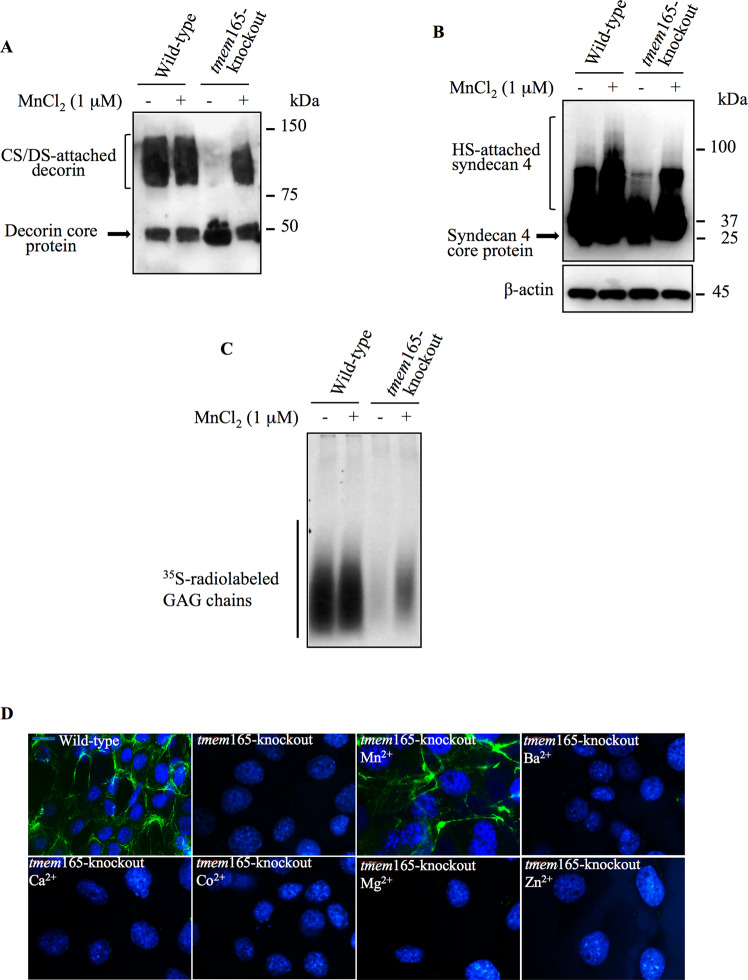
Manganese supplementation rescue GAG elongation in *TMEM*165-deficient cells. **A** Detection of decorin in conditioned medium of wild-type and *tmem*165-knockout mouse ATDC5 cells transfected with the expression vector for decorin. (**B**) Detection of HA-tagged syndecan 4 in cell lysates of wild-type and *tmem*165-knockout mouse ATDC5 cells transfected with HA-tagged syndecan 4 expression vector and grown in the presence or absence of MnCl_2_ (1 µM). β-actin was used as loading control (*n* = 3). **C** SDS-PAGE and autoradiography of [^35^S]-sulfate radiolabelled GAG chains primed with 4MU-Xyl in wild-type and *tmem*165-mutant mouse ATDC5 cells grown in the presence or absence of MnCl_2_ (1 µM) (*n* = 3). One representative blot of three independent experiments is shown. **D** Immunofluorescence analysis of cell surface HSGAG chains using anti-HS specific antibodies (green) in wild-type and *tmem*165-knockout mouse ATDC5 cells cultured in the absence and presence of Mn^2+^, Ba^2+^, Ca^2+^, Co^2+^, Mg^2+^ and Zn^2+^. The nucleus was stained with DAPI (blue). Digital images were captured with an inverted microscope, Leica DM13000. Representative images from three independent experiments are shown. Scale bar: 50 μm.

It has been established that Mn^2+^ participates in the catalytic activity of various Golgi glycosyltransferases including CS and HS polymerizing enzymes [10, 11]. To determine whether defects in the elongation of CS- and HS-GAG chains in *tmem*165-knockot cells is due to defect in Mn^2+^ homeostasis, cells were cultured in the absence or presence of 1μM of Mn^2+^. Western blot analysis of decorin produced in wild-type mouse ATDC5 cells showed similar pattern either in the absence or presence of Mn^2+^. However, decorin expressed in *tmem*165-knockout cells that were cultured in the presence of Mn^2+^ was larger in size, compared to that produced in the absence of Mn^2+^ and exhibit similar pattern as decorin in wild-type (Fig. 4A). These data revealed that supplementation of the culture medium with Mn^2+^ rescues elongation of decorin GAG chains in *tmem*165-knockout cells. As polymerization of HS-GAG chains are also impaired in *tmem*165-knockout cells, we investigated whether Mn^2+^ is able to restore the polymerization of HS-GAG chains. Remarkably, supplementation of Mn^2+^ in the culture medium of *tmem*165-knockout cells resulted in the synthesis of HA-syndecan 4 with higher molecular weight compared to that produced in the absence of Mn^2+^ (Fig. 4B), indicating that elongation HS-GAG chains attached to HA-syndecan 4 is restored when Mn^2+^ is supplied in the culture medium. To further confirm that Mn^2+^ rescues GAG chains elongation in *tmem*165-knockout cells, 4MU-Xyl was used as exogenous substrate to monitor the synthesis of GAG chains. Wild-type and *tmem165*-mutant mouse ADTC5 cells were cultured in the presence or absence of Mn^2+^ along with 4MU-Xyl primer and [^35^S]-sulfate to radiolabel newly synthesized GAG chains. SDS-PAGE analysis of radiolabelled 4MU-Xyl-primed GAG chains showed that in the absence or presence of Mn^2+^, high amount of GAG chains was produced in wild-type cells. In *tmem*165-knockout cells, a very week signal was observed in the absence of Mn2^+^ supplementation, whereas a significant amount of elongated GAG chains was observed when cells were supplemented with Mn^2+^ (Fig. 4C). Altogether, these data indicate that Mn^2+^ rescues the polymerization of both CS- and HS-GAG chains in *tmem*165-knockout cells and suggest that the activity of GAG polymerizing enzymes is impaired in *tmem*165-knockout cells due to disturbed Golgi Mn^2+^ homeostasis.

To examine whether the addition of other ions could rescue the blockage in elongation of glycosaminoglycan chains, *tmem*165-knockout cells were cultured in presence of Mn^2+^, Ba^2+^, Ca^2+^, Co^2+^, Mg^2+^ and Zn^2+^, respectively and endogenous cell surface HS-GAG chains were analyzed by indirect immunofluorescence using anti-HS monoclonal antibody 10E4, which is commonly used to detect HS chains of PGs. Prominent staining of the cell surface HS-GAGS was observed in mouse ATDC5 control cells, whereas no staining could be observed in *tmem*165-knockout mouse ATDC5 cells (Fig. 4D) indicating that the knockout of *tmem*165 prevented the synthesis of cell surface HS-GAG chains. Interestingly, supplementation of cell culture medium of *tmem*165-knockout ADTC5 cells with Mn^2+^ rescued the synthesis of HS-GAG chains, whereas supplementation with divalent ions Ba^2+^, Ca^2+^, Co^2+^, Mg^2+^ or Zn^2+^ did not (Fig. 4D), indicating that among the divalent ions tested only Mn^2+^ is able to restore defects induced by loss of TMEM165 on HS-GAG chain elongation.

### Ca^2+^/calmodulin-dependent protein kinase IIα pathway is activated in TMEM165-deficient cells

**Fig. 5:**
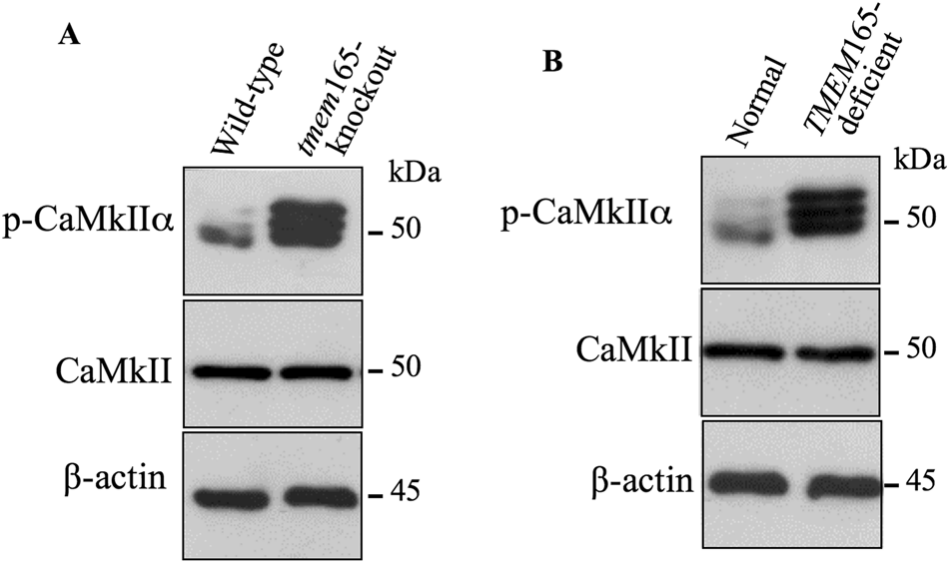
Phospho-CaMKIIα is activated in *TMEM*165-deficient cells. **A** Immunoblot analysis of phospho-CaMkIIα (pCaMkIIα) level in cell lysates of wild-type and *tmem*165-knockout mouse ATDC5 cells and (**B**) in cell lysates of normal fibroblasts and *TMEM*165-deficient fibroblasts from CDG patients, using specific antiphospho-CaMkIIα and anti-CaMkIIα antibodies. β-actin was used as loading control (*n* = 3). Representative images from three independent experiments are shown.

Direct implication of TMEM165 in the Golgi transport of either Ca^2+^ or Mn^2+^ is not yet been reported. However, it is well known that Ca^2+^/calmodulin-dependent protein kinase II (CaMkII) is activated when intracellular concentration in [Ca^2+^]_i_ is elevated. Analysis of the phosphorylation status of CaMkIIα in *tmem*165-knockout and wild-type mouse ATDC5 cells revealed that the level of phospho-CaMkIIα (pCaMkIIα) was strongly increased in *tmem*165-knockout cells, compared to wild-type cells (Fig. 5A) indicating that CaMkIIα was activated in TMEM165-knockout cells. Likewise, the phosphorylation level of CaMkIIα was remarkably increased in *TMEM165*-deficient patient fibroblast cells, compared to normal fibroblasts (Fig. 5B). Altogether, these data indicated that the loss of TMEM165 induces defects in both Ca^2+^ and Mn^2+^ homeostasis and support the notion that TMEM165 is probably involved in the regulation of both Ca^2+^ and Mn^2+^ homeostasis.

### TMEM165 deficiency impairs TGF-β and BMP signaling pathways

**Fig. 6:**
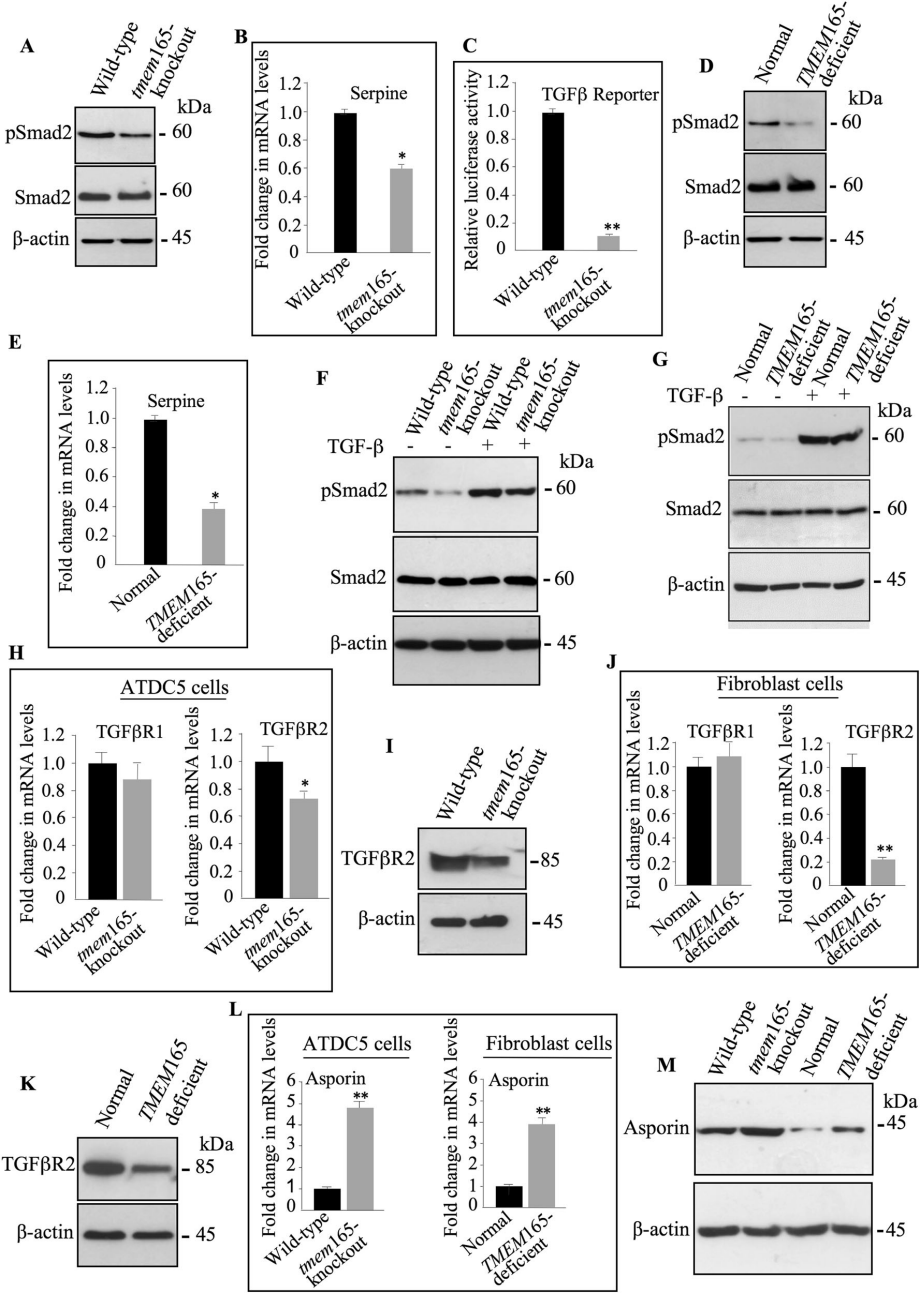
TGF-β signaling pathway is impaired in *TMEM*165-deficient cells. **A** Detection of phosphorylated Smad2 (pSmad2) and total Smad2 (Smad2) in cell lysates from wild-type and *tmem*165-knockout mouse ATDC5 cells (*n* = 3). **B** Fold changes of serpine expression in *tmem*165-knockout cells normalized to wild-type mouse ATDC5 cells. **C** Fold changes of TGF-β reporter activity in *tmem*165-knockout cells normalized to wild-type mouse ATDC5. **D** Detection of phosphorylated Smad2 (pSmad2) and total Smad2 in cell lysates from control and *TMEM*165-deficient CDG patient fibroblast cells (*n* = 3). **E** Fold changes of serpine expression in *TMEM*165-deficient fibroblasts normalized to normal fibroblast cells. **F** Detection of p-Smad2 in cell lysates from wild-type and *tmem*165-knockout mouse ATDC5 cells and (**G**) from normal and *TMEM*165-deficient CDG patient fibroblast cells treated or not with TGFβ1 (1 ng/ml) for 1 hour (*n* = 3). **H** Fold changes of TGFβR1 and TGFβR2 expression in *tmem*165-knockout mouse ATDC5 cells normalized to wild-type mouse ATDC5 cells. **I** Detection of TGFβR2 in cell lysates from wild-type and *tmem*165-mutant mouse ATDC5 (*n* = 3). **J** Fold changes of TGFβR1 and TGFβR2 expression in *TMEM*165-deficient fibroblasts from CDG patient normalized to normal fibroblast cells. **K** Detection of TGFβR2 in cell lysates from normal fibroblasts and *TMEM*165-deficient CDG patient fibroblast cells (*n* = 3). **L** Fold changes of asporin expression in *tmem*165-knockout cells normalized to wild-type mouse ATDC5 cells and in *TMEM*165-deficient fibroblast cells normalized to normal fibroblast cells. **M** Detection of asporin in conditioned medium of wild-type and *tmem*165-knockout mouse ATDC5 cells and of normal fibroblasts and *TMEM*165-deficient CDG patient fibrobast cells. β-actin was used as loading control (*n* = 3). qPCR values were normalized for the housekeeping gene ribosomal protein S29 and are expressed as the relative expression compared with control. Data are expressed as mean ± S.D. Statistical analysis was performed with an unpaired Student’s *t* test (*n* = 3; *p < 0.05; **p < 0.01). Representative images from three independent experiments are shown.

As a number of human genetic mutations causing a wide range of inheritable diseases of skeletal development are related to TGF-β and BMP signaling [12] and owing to the key role of GAG chains in the regulation of several signaling pathways, we sought to investigate whether loss of TMEM165 affects TGF-β/BMP signaling pathways. Interestingly, Western blot analysis showed that phospho-Smad2 level was reduced in *tmem*165-knockout cells, compared to wild-type (Fig. 6A). In addition, the mRNA levels of the serpine 1 (PAI-1) gene, which is positively correlated with TGF-β signaling activation were significantly lower in cells knocked down for *tmem*165 (Fig. 6B), therefore indicating that the loss of TMEM165 perturbs TGF-β signaling in mutant cells. These data were further confirmed by using the p(CAGA)12-luc TGF-β reporter plasmid which showed that the luciferase activity was markedly (7-fold) lower in mutant cells, compared to wild-type cells confirming that the TGF-β/Smad axis is functionally impaired in *tmem*165-knockout cells (Fig. 6C). Similar results were obtained in *TMEM*165-deficient patient fibroblast cells. Indeed, phospho-Smad2 level was reduced in *TMEM*165-deficient fibroblasts compared to normal fibroblasts (Fig. 6D) and the expression of serpine1 gene showed significant (2.5-fold) downregulation (Fig. 6E). Therefore, indicating that TMEM165 deficiency impairs the expression of the TGF-β/Smad downstream responsive genes in *TMEM*165-deficient fibroblasts.

To determine whether response to TGF-β stimulation is affected in mutant cells, phosphorylation level of Smad2 in response to TGF-β was examined. Treatment of *tmem*165-knockout mouse ATDC5 cells with TGF-β induced the phosphorylation of Smad2, however the level of phospho-Smad2 is lower compared to that observed in wild-type cells (Fig. 6F), indicating that mutant cells were less responsive to TGF-β than wild-type cells. Likewise, stimulation of *TMEM*165-deficient and normal fibroblasts with TGF-β induced less phosphorylation of Smad2 in mutant fibroblasts, compared to normal fibroblasts (Fig. 6G). Altogether, these results demonstrate that both the baseline and TGF-β-induced levels of phospho-Smad2 were significantly reduced in mutant cells, therefore revealing for the first time that TMEM165 deficiency functionally impaired the TGF-β/Smad2 signaling axis.

To identify the mechanism involved in the alteration of the TGF-β/Smad signaling pathway, we investigated the mRNA expression levels of TGF-β receptors, TGFβR1 and TGFβR2 in *tmem*165-knockout and wild-type mouse ATDC5 cells. As shown in Fig. 6H, a significant decrease in mRNA levels of TGFβR2 was observed in *tmem*165-mutant mouse ATDC5 cells, compared with wild-type cells. Consistent with RT-qPCR data, significant reduction in the protein level of TGFβR2 was observed in *tmem*165-knockout mouse ATDC5 cells, compared to wild-type cells (Fig. 6I). Similarly, fibroblast cells of *TMEM*165-deficient patient showed strong decrease in the mRNA levels (Fig. 6J) and protein expression of TGFβR2 (Fig. 6K), compared to normal cells. Downregulation of TGF-β signaling may also result from up-regulation of negative regulators such as asporin, an extracellular protein that suppresses TGF-β signaling by direct interaction with TGF-β thus preventing its binding to TGFβR2. Analysis of mRNA levels of asporin (*Aspn*) in *tmem*165-knockout mouse ATDC5 cells and in *TMEM*165-deficient patient fibroblasts showed a remarkable increase (4-fold) in mutant cells compared to normal cells (Fig. 6L). Assessment of asporin protein expression showed higher levels of expression in *tmem*165-deficient mouse ATDC5 and *TMEM*165-mutant fibroblast cells, compared to normal cells (Fig. 6M). Altogether, these data support the notion that impaired TGF-β signaling in *TMEM*165-deficient cells is associated with downregulation of the expression of TGFβR2 receptor and upregulation TGF-β antagonist, asporin.

**Fig. 7:**
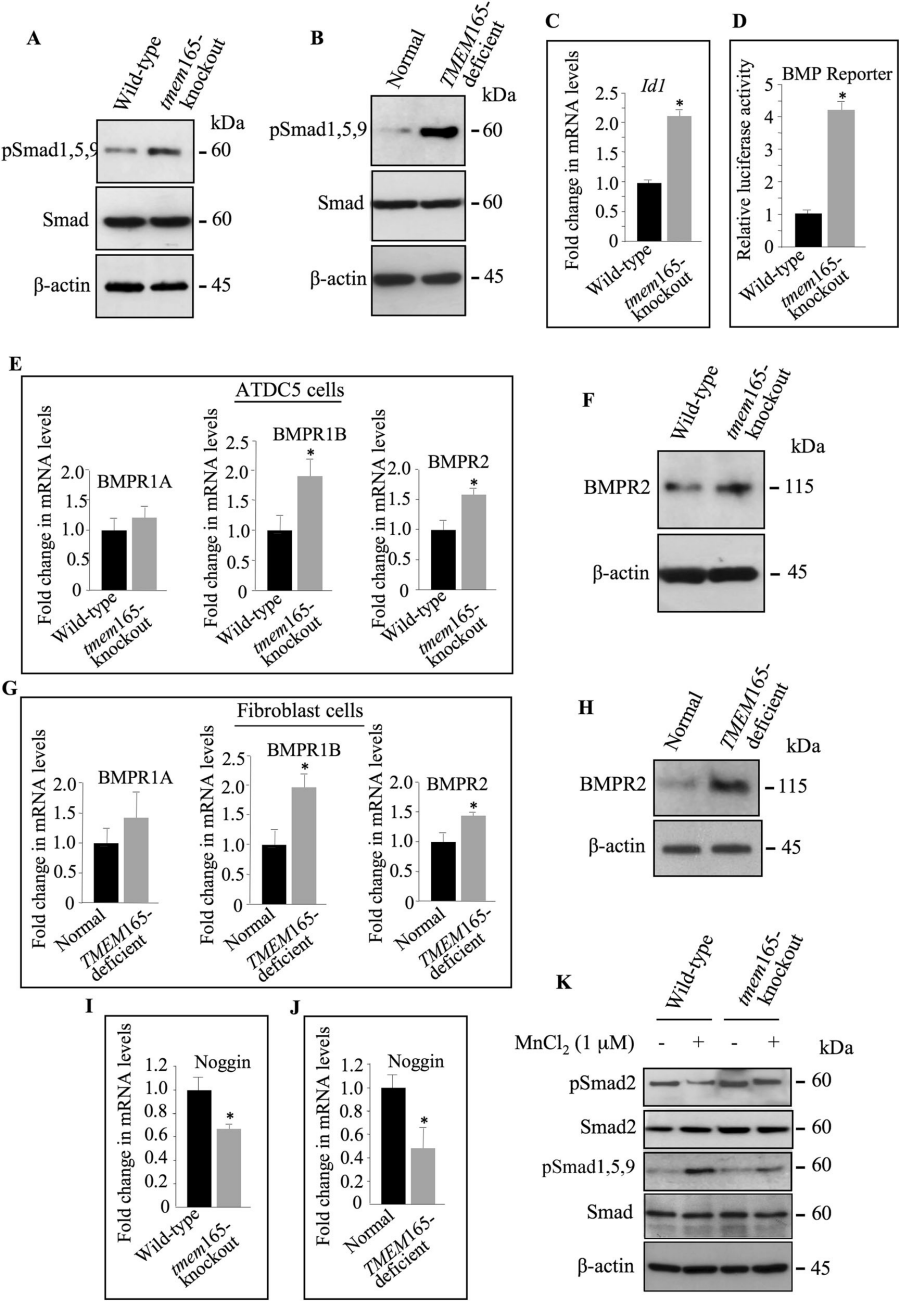
BMP signaling is activated in *TMEM*165-deficient cells. **A** Detection of phosphorylated Smad1, 5, 9 (pSmad1,5,9) and total Smad in cell lysates from wild-type and *tmem*165-mutant mouse ATDC5 cells and (**B**) from normal fibroblasts and *TMEM*165-deficient CDG patient fibroblast cells (*n* = 3). **C** Fold changes of Id1 expression in *tmem*165-knockout cells normalized to wild-type mouse ATDC5 cells. **D** Fold changes of BMP reporter activity in *tmem*165-knockout cells normalized to wild-type mouse ATDC5 cells. **E** Fold changes of BMPR1A, BMPR1B and BMPR2 expression in *tmem*165-knockout cells normalized to wild-type mouse ATDC5 cells. **F** Detection of BMPR2 in cell lysates of wild-type and *tmem*165-knockout mouse ATDC5 cells. β-actin was used as loading control (*n* = 3). **G** Fold changes of BMPR1A, BMPR1B and BMPR2 expression in *TMEM*165-deficient fibroblasts normalized to normal fibroblast cells. **H** Detection of BMPR2 in cell lysates of normal fibroblasts and *TMEM*165-deficient CDG patient fibroblast cells. β-actin was used as loading control (*n* = 3). **I** Fold changes of Noggin expression in *tmem*165-knockout mouse ATDC5 cells normalized to wild-type ATDC5 cells and (**J**) in *TMEM*165-deficient CDG patient fibroblasts normalized to normal fibroblast cells. qPCR values were normalized for the housekeeping gene ribosomal protein S29 and are expressed as the relative expression compared with control. Data are expressed as mean ± S.D. Statistical analysis was performed with an unpaired Student’s *t* test (*n* = 3; *p < 0.05; **p < 0.01). **K** Detection of phosphorylated Smad2 (pSmad2) and Smad1, 5, 9 (pSmad1,5,9) and of total Smad in cell lysates from wild-type and *tmem*165-mutant mouse ATDC5 cells cultured in medium with or without Mn^2+^ supplementation. β-actin was used as loading control (*n* = 3). Representative images from three independent experiments are shown.

BMP signaling plays a key role in skeletal development and alterations in BMP signaling pathway are major underlying cause of human skeletal disorders [13]. To determine whether TMEM165 deficiency affects BMP signaling, we examined the phosphorylation status of Smad 1,5,9 downstream mediators of BMP receptor activation. Interestingly, immunoblot analysis showed that the level of phospho-Smad 1,5,9 (pSmad1,5,9) was higher in *tmem*165-knockout mouse ATDC5 cells (Fig. 7A) and in *TMEM*165-deficient patient fibroblast cells (Fig. 7B). Analysis of the mRNA levels of *Id1* gene, a BMP downstream target gene, showed two-fold increase in *tmem*165-knockout cells, compared to wild-type cells (Fig. 7C). To further confirm the increase of BMP signaling in *tmem*165-knockout cells, the vector pGL3-BRE-Luc containing a BMP responsive promoter element (BRE) was used to transfect wild-type and *tmem*165-mutant mouse ATDC5 cells. As shown in Fig. 7D, analysis of luciferase activity showed a significant increase (4-fold) in *tmem*165-knockout ATDC5 cells, compared to wild-type cells thus bringing evidence that BMP signaling is increased in *tmem*165-mutant cells. In an attempt to identify the mechanism involved in the alteration of the BMP/Smad signaling pathway, we investigated the mRNA levels of type I and type II BMP receptors *ie*. BMPR1A, BMR1B and BMPR2 in *TMEM*165-deficient and wild-type cells. As shown in Fig. 7E, mRNA levels of BMP receptors were increased in *tmem*165-knockout mouse ATDC5 cells, compared to wild-type cells. The increased expression of BMPR2 at the protein level in mutant cells was confirmed by Western blot analysis (Fig. 7F). Similar results were observed, either for mRNA expression levels of *BMPR1A*, *BMR1B* and *BMPR2* (Fig. 7G) or for BMPR2 protein expression (Fig. 7H) in *TMEM*165-deficient patient fibroblast cells, compared to normal fibroblast cells. In addition, analysis of the mRNA levels of noggin, a BMP ligand antagonist, showed strong reduction in *tmem*165-deficient mouse ATDC5 cells (Fig. 7I) and in *TMEM*165-deficient patient fibroblasts (Fig. 7J), compared to normal cells. These data suggest that upregulation of BMP receptors and downregulation of BMP antagonist may account for the increased BMP signaling observed in *TMEM*165-deficient cells. Overall, this study showed that TMEM165 deficiency functionally impairs TGFβ/BMP signaling pathways in *TMEM*165-deficient cells.

We next analyzed whether supplementation with Mn^2+^ rescued TGFβ/BMP signaling. Interestingly, Western blot analysis showed that phospho-Smad2 and phospho-Smad 1,5,9 levels were restored to normal levels in *tmem*165-knockout mouse ATDC5 cells when cultured in the presence of Mn^2+^ (Fig. 7K), indicating that supplementation with Mn^2+^ restored TGFβ/BMP signaling in *tmem*165-knockout cells.

### TMEM165 deficiency promotes early chondrocyte differentiation and hypertrophy

**Fig. 8:**
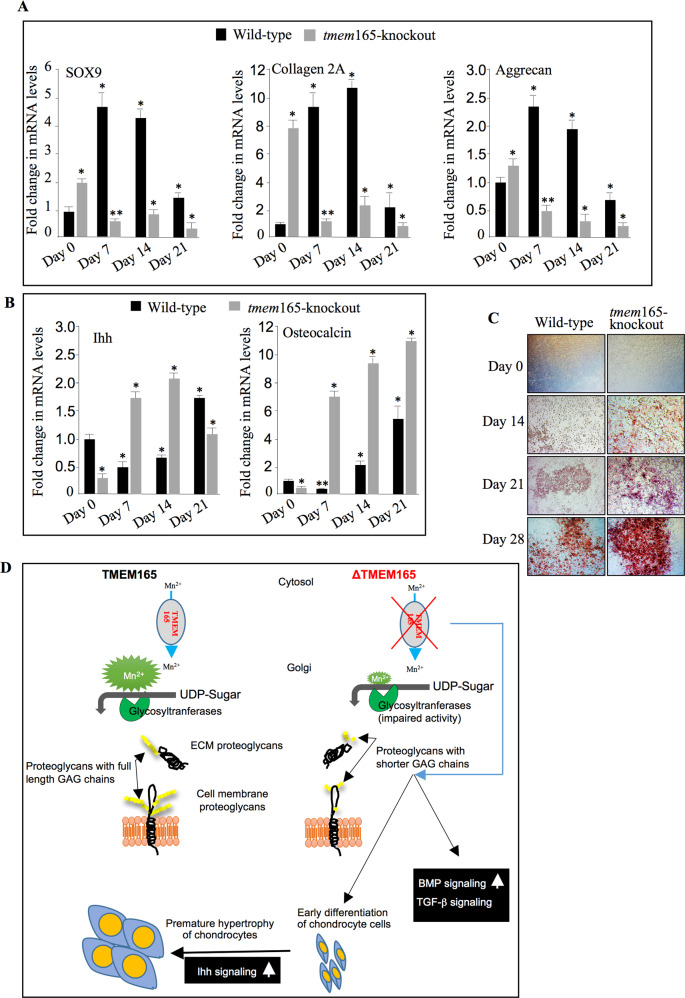
Early hypertrophic differentiation of *tmem*165-deficient mouse ATDC5 cells. **A** Fold changes of chondrogenic markers expression in wild-type mouse ATDC5 cells and *tmem*165-knockout cells. RT-qPCR analysis of the mRNA levels of chondrogenic markers SOX9, Col2A, and Aggrecan, and (**B**) of hypertrophic markers Ihh and OCN. qPCR values were normalized for the housekeeping gene ribosomal protein S29 and are expressed as the relative expression compared with control. Data are expressed as mean ± S.D. Statistical analysis was performed with an unpaired Student’s *t* test (*n* = 3; *p < 0.05; **p < 0.01). **C** Mineralization of wild-type and *tmem*165-deficient mouse ATDC5 cells analyzed by Alizarin red staining at Days 0, 14, 21, and 28. Representative images from three independent experiments are shown. **D** Loss of TMEM165 function impairs Golgi Mn^2+^ homeostasis necessary for glycosyltransferase polymerization activities leading to blockage in the elongation of GAG chains of proteoglycans. Blockage in the elongation of GAG chains by loss of TMEM165 may account for dysregulation of TGF/BMP and Ihh signaling, and therefore in defects in chondrocyte differentiation and maturation. However, other mechanisms can’t be ruled-out.

Chondrogenesis process is tightly regulated during skeletal development and alterations in the chondrocyte maturation result in defects in endochondral bone development. To investigate whether chondrogenic differentiation of prechondrocyte mouse ATDC5 cells is affected in the absence of TMEM165, wild-type and *tmem*165-knockout mouse ATDC5 cells were induced by insulin into chondrogenic differentiation and the mRNA levels of chondrogenic markers including Sox9, Col2a1, AGN, Ihh, and OCN were measured by RT-qPCR before induction and at seven, forteen and twenty-one days post induction. The results showed that before induction (day 0), mouse ATDC5-knockout cells express higher mRNA levels of the chondrogenic markers Sox9, Col2a1, and AGN and lower mRNA levels of pre-hypertrophic and hypertrophic markers Ihh and OCN, compared to wild-type cells (Fig. 8A, B (day 0)). These results indicate that loss of TMEM165 expression induces early chondrogenic differentiation of prechondrogenic mouse ATDC5 cells. Importantly, analysis of the expression of the markers at seven days post induction revealed that *tmem*165-deficient mouse ATDC5 cells undergo rapid maturation and hypertrophy as evidenced by downregulation of chondrogenic markers Sox9, Col2a1, AGN and upregulation of pre-hypertrophic and hypertrophic markers Ihh and OCN. The expression of hypertrophic markers still increases at 14 and 21 days. In contrast, as expected, in wild-type cells the chondrogenic markers were up-regulated at 7 and 14 days post induction, and pre-hypertrophic and hypertrophic markers were downregulated. However, at 21 days post induction chondrogenic markers were down-regulated, and pre-hypertrophic and hypertrophic markers were up-regulated (Fig. 8A, B).

We also observed an early mineralization in *tmem*165-deficient mouse ATDC5, compared to wild-type cells. Indeed, mineralization was detected in *tmem*165-deficient mouse ATDC5 at day 14 after initiation of differentiation, whereas it appeared only after 21 days of differentiation in wild-type cells (Fig. 8C). Altogether, these data bring evidence that TMEM165 deficiency promotes premature chondrocyte maturation and hypertrophy, a process which affects endochondral ossification and may lead to dwarfism.

## Discussion

Here, we generated *tmem*165-knockout pre-chondrocyte mouse ATDC5 and HEK293 cells and showed that loss of TMEM165 led to strong defects in the synthesis of PGs. Decrease content of PGs in cartilage was also reported in *tmem* 165-deficient zebrafish [14]. Importantly, we showed a dramatical reduction in the length of HS- and CS-GAG chains, revealing for the first time that elongation of PG-GAG chains is impaired in TMEM165 deficient cells. We showed that overexpression of CS elongating enzymes CHSY1 and CHSY2 did not rescue the elongation of CS-GAG chains; however, Mn^2+^ supplementation restores the elongation of CS- and HS GAG chains of PGs in *tmem*165-knockout cells. Golgi glycosyltransferases use UDP-sugars as a donor substrate and require Mn^2+^ at their catalytic site to be fully active. These findings revealed that TMEM165 plays an essential role in the synthesis of PGs by regulating homeostasis of Mn^2+^ in the Golgi compartment. We can therefore hypothesize that GAG chains are aborted because of lack of sufficient pool of the cofactor Mn^2+^ in the Golgi necessary for the synthesis of normal sized GAG chains. How Mn^2+^ supplementation rescues GAG chains elongation is not known, however we previously showed that the rescue of Golgi *N*-glycosylation defects in *TMEM*165-deficient cells by extracellular Mn^2+^ involves a transit across the ER compartment. Mn^2+^ enters *TMEM165*-deficient HEK293 cells through plasma membrane transporters and reaches the ER compartment by a mechanism involving SERCA pumps then transported from the ER to the Golgi probably via SPCA pumps or other yet unknown mechanism [15].

Deficiency of Mn^2+^ results in birth defects including abnormal or poor bone formation and susceptibility to seizures [16]. Mutations in SLC39A8 a plasma membrane protein able to transport Mn^2+^ are characterized by low blood levels of Mn^2+^ associated with delayed development, dwarfism, and profound psychomotor retardation [17].

Analysis of BMP and TGF-β signaling pathways revealed that they are functionally impaired in *TMEM*165-deficient cells. TGF-βs and BMPs play an important role in several stages of chondrogenesis. It has been reported that TGF-β inhibits the terminal differentiation of chondrocytes in high-density chondrocyte pellets or long bone cultures in vitro [18]. We found that TMEM165 deficiency led to downregulation of TGF-β signaling associated with downregulation of TGFβRII receptor and upregulation of TGF-β antagonist, asporin. We showed that the TGF-β signaling was activated when the cells were exogenously treated with TGF-β1 but at lower extent compared to wild-type cells, indicating that both basal and inducible TGF-β signaling activation is impaired in *TMEM*-165-deficient cells. TGF-β activates Smad2 and Smad3 through binding to TGFβRII receptor and recruitment of ALK5 receptor (TGFβRI) [19]. Noteworthy, mice lacking Tgfβr2 or Alk5 in chondrocytes exhibit skeletal defects [20] and inhibition of TGFβ signaling induces chondrocyte hypertrophy [12].

We have analyzed the BMP signaling pathway and found that phospho-Smad1,5,9 was upregulated in *TMEM*165-deficient cells, compared to normal cells. Investigation of the mechanisms involved revealed that the BMP receptors BMPR2 and BMPR1B are upregulated and the BMP antagonist noggin is downregulated in *TMEM*165-deficient cells. Noteworthy, BMP regulates longitudinal growth and excessive BMP signaling has been shown to accelerate chondrogenesis and chondrocyte differentiation [21, 22]. Brachydactylies type 2 (BDB2) is a *NOG* mutation characterized by absence of terminal structures of the toes and digits due to excessive BMP signaling [23]. Mutations in *NOG* cause inability of the antagonist to bind BMP or heparin and to sequester BMPs in ECM which results in excessive BMP signaling [24, 25].

The increased levels of hypertrophic marker Ihh and osteocalcin in *tmem*165-knockout prechondrocyte mouse ATDC5 cells during differentiation lead to early hypertrophy of chondrocytes. Indeed, overexpression of Ihh induces early chondrocyte hypertrophy and ossification during chondrogenesis. It has been suggested that TGFβ inhibits hypertrophy induced by Ihh [26]. Our study has shown that TGF-β signaling is downregulated and Ihh is upregulated in *tmem*165-mutant mouse ATDC5 cells, suggesting accelerated chondrocyte hypertrophy. Interestingly, we found that loss of TMEM165 induced early mineralization in ATDC5 cells. Premature transition of the chondrocyte phenotype from proliferating to hypertrophic chondrocytes and the early onset of mineralization may trigger a premature replacement of cartilage by bone leading to defects in skeletal development and hence to dwarfism.

As summarized in Fig. 8D, our findings indicate that TMEM165 deficiency causes abnormalities in PG synthesis and aberrant signaling resulting in early chondrocyte maturation and hypertrophy. PGs and their GAG chains are key components of the ECM and are involved in the organization and function of the matrix. Also, alterations in the structure of GAG chains may induce profound changes in the organization and function of the ECM and hence in cell-matrix interactions and signaling which may lead to defects in endochondral ossification.

## Materials and methods

### Cell culture and treatments

Skin fibroblast cells were derived from skin biopsy specimens from healthy controls and *TMEM165*-deficient patients as described by Foulquier et al, 2012 and were maintained in Eagle’s minimum essential medium in humidified 37 °C incubator. Culture medium was supplemented with 10% fetal bovine serum and 1% combination of 100 U penicillin/0.1 mg/ml streptomycin. Mouse ATDC5 cells (Riken cell RCB0565, Tsukubai, Japan) were cultured in DMEM-F12 complete medium (2 mM glutamine, 100 μg/ml streptomycin, 100 IU/ml penicillin, and 5% (v/v) foetal bovine serum) and HEK293 cells (ATCC CRL-3216, LGC Standards, France) were cultured in DMEM complete medium with 10% (v/v) foetal bovine serum at 37 °C in a humidified atmosphere supplemented with 5% CO_2_. Cells were seeded onto six-well plates at 2 × 10^5^ cells/well and allowed to attach overnight in standard culture conditions. For treatment with Mn^2+^, mouse ATDC5 cells were cultured in DMEM F12 complete medium until reaching 80% confluency then the medium was replaced with DMEM-F12 without FBS and containing 1 μM of divalent ions, for 36 h. For treatment with growth factors, cells were cultured in DMEM F12 complete medium until reaching 80% confluency then the medium was replaced with DMEM-F12 without FBS and containing 1 ng/ml of TGF-β1 (R&D Systems, Minneapolis, MN USA) or vehicle (0.1% BSA in PBS) for 1 h. Cells were then washed twice with PBS and stored at -80 °C prior to protein or gene expression analyses. For chondrogenic differentiation, mouse ATDC5 cells were seeded in 12 well/plate at 4 × 10^4^ cells/well and cultured in DMEM-F12 complete medium containing 50 μg/ml human transferrin and 3 × 10^–8^ M sodium selenite (Sigma, Saint Louis, MO) until confluency (day 0), then chondrogenesis was induced by addition of 100 μg/ml of human insulin (Sigma, Saint Louis, MO) in the culture medium. The medium was replaced every second or third day. Cells were washed twice with PBS and stored at −80 °C prior to protein or gene expression analyses. For Alizarin red staining, cells were fixed in ethanol for 30 min then washed with PBS and 1% Alizarin red solution (Sigma, pH 4.2) was added to the cell layers for 15 min at room temperature. Cells were washed with distilled water and images were captured.

### Gene expression analysis

Total RNA from cells was extracted using TRIzol (Lifetech, Carlsbad, CA) and purified with RNeasy kit (Qiagen, Hilden, Germany) according to manufacturer’s instructions. The reverse transcription was performed using 500 ng of total RNA from each sample with iScript Ready to use cDNA supermix (BIO-RAD, Hercules, CA). Quantitative PCR was performed with iTaq™ Universal SYBER Green Supermix kit (BIO-RAD, Hercules, CA) using StepOnePlus™ Real-Time PCR Systems (Applied Biosystems, Foster city, CA) and validated primers.

### Metabolic labeling of GAG chains

Metabolic labeling of GAG chains of PGs was carried out using [^35^S]-sulfate incorporation method as described by [27]. Briefly, subconfluent mouse ATDC5 cells grown in six-well culture plate were radiolabeled with 10 µCi/ml of [^35^S]-sulfate (Perkin Elmer, Courtabœuf, France) overnight. Then, conditioned culture medium was collected, digested with papain (1 mg/ml), and [^35^S]-labeled GAGs were precipitated by cetylpyridinium chloride (CPC) as described by [28]. When GAG chains were primed by 4MU-Xyl, cells were cultured in the presence of 10 µCi/ml of [^35^S]-sulfate and 100 μM of 4MU-Xyl overnight, and radiolabeled GAG chains were directly precipitated from conditioned medium by CPC. The CPC precipitated radiolabeled GAGs were separated by SDS-PAGE on a 4–20% Tris/Glycine gel. The gel was dried and exposed to autoradiography film.

To measure the rate of sulfate incorporation into GAG chains of PGs, mouse ATDC5 cells were radiolabeled with 10 µCi/ml of [^35^S]-sulfate for 6 h then, conditioned culture medium was collected and digested with papain (1 mg/ml). [^35^S]-labeled GAG chains were precipitated by CPC dissolved in solvable and mixed in scintillation fluid (Perkin Elmer, MA, USA). The radioactivity associated with GAGs was measured by liquid scintillation counting (Packard, Rungis, France).

### Indirect immunofluorescence staining

The mouse ATDC5 cells were grown on glass coverslips and fixed with 4% (w/v) paraformaldehyde in PBS for 20 min. Cells were permeabilized by treatment with 0.1% (w/v) Triton X-100/PBS solution for 4 min. After extensive washing in 0.2% (w/v) fish skin gelatin in PBS, cells were then incubated with primary antibodies anti-TMEM165 (Cat# HPA038299, 1:100, Atlas Antibodies) or anti-HS (Cat# 370255-1, 1:100, AMSBIO) for 20 min. Cells were washed several times in 0.2% (w/v) fish skin gelatin in PBS and incubated with secondary antibodies coupled with Alexa Fluor 488 (Cat# A-21206 or Cat# A-11017, Molecular Probes) for 20 min. Cells were washed with 0.2% (w/v) fish skin gelatin in PBS and incubated with primary antibodies anti-GM130 (Cat# 610822, 1:100, BD Biosciences) for 20 min. Cover slips were then washed several times in 0.2% (w/v) fish skin gelatin in PBS and incubated with secondary antibodies coupled with Alexa Fluor 555 (Cat# A-21428, Molecular Probes) for 20 min. Cells were washed with PBS and nuclei were stained with Hoechst/PBS solutions then coverslips were mounted with Moviol (National Diagnostics, U.K.) containing 1% propylgallate (Sigma, Saint Louis, MO). Digital images were captured with an inverted microscope Lieca DMI3000 B (Leica Microsystems, Germany).

### CRISPR/Cas9 mutation of TMEM165 gene

Sense 5ʹ**CACC**GCTATAACCGGCTGACTGTGC3ʹ and antisense 5ʹ**AAAC**GCACAGTCAG CCGGTTATAGC3ʹ oligonucleotides (1 μg) containing 20 bp sequence (underlined) targeting TMEM165 exon 2 and cohesive ends (bold) with the vector were annealed in annealing buffer (60 mM Tris-HCl, pH7.5; 500 mM NaCl; 60 mM MgCl_2_; 10 mM DTT) and ligated into BbsI sites of pUC57-attbU6 sgRNA vector. This vector is a basic vector with U6 promoters and improved Cas9 binding sites. 1 μg of pUC57-TMEM165, 100 ng of SVneo vector (that confer resistance to geneticin), and 1 μg of pSpCas-9 vector was used to transfect mouse ATDC5 cells grown at 80% confluency in six wells/plate, using Lipofectamine 2000© (Invitrogen, Carlsbad, CA) according to the instructions of the supplier. 24 h after transfection, the medium was replaced by medium containing 0.2 mg/ml geneticin sulfate and cells were cultured for 48 h before trypsinized and cloning by serial dilution in 96 wells/plate. Several clones were obtained and amplified in six wells/plates and analyzed for the presence of mutations in the targeted sequence by PCR amplification and sequencing of the genomic DNA flanking the targeted region. *TMEM*165-knockout HEK293 cells were generated using CRISPR/cas9 technique and were previously described by Morelle et al, 2017.

### Plasmids and transfection

Chsy1, Chsy2, Decorin, and HA-syndecan 4 cDNAs were generated by PCR and cloned into EcoRI and BamHI or SmaI and PstI sites of pCMV empty vector (Stratagene, Valencia, CA). Decorin-S34A mutant was generated by site-directed mutagenesis using QuikChange XLII (Agilent, CA, USA) according to the manufacturer’s instructions. For transfection, cells were seeded in 6-well culture plate until 80% confluency and transfected with 1 µg of either pCMV-Decorin, pCMV-HA-Syndecan 4, pCMV-Decorin-S34A, pCMV-Myc-Chsy1, pCMV-HA-Chsy2, or pCMV-empty vector using lipofectamine 2000 transfection reagent (Invitrogen, Carlsbad, CA) according to manufacturer’s instructions. Expression of decorin in culture medium and of syndecan 4 in cell lysate was analyzed at 48 h post transfection by Western blotting.

### TGF-β and BMP luciferase reporter activity assays

p(CAGA)12-luc and pGL3-BRE-Luc Wild-type and *tmem*165-knockout mouse ATDC5 cells were plated onto twenty-four-well plates and grown to 80% confluency. Cells were transfected with 500 ng of p(CAGA)12-luc and pGL3-BRE-Luc promoter constructs, respectively along with 25 ng of pRL-TK vector (Promega, Madison, WI) using. Twenty-four hours after transfection, Firefly and Renilla luciferase activities in cells of each well were measured with the Dual-Luciferase Assay System (Promega) using a Berthold (Bad Wildbad, Germany) luminometer. Luciferase activities were normalized to pRL-TK vector activity.

### Western blotting

Total protein from cells was extracted using RIPA buffer (150 mM NaCl, 50 mM Tris-HCl, pH 7.5, 1% deoxycholate, 0.1% SDS, 1% Triton X-100) supplemented with protease and phosphatase inhibitors (Roche Diagnostics, Indianapolis, IN, USA). Cell lysates were sonicated on ice and protein concentration of the samples was determined by the Bradford method. Proteins (50 μg/lane) were separated on 10% SDS-PAGE gels, transferred to a PVDF membrane (Millipore, Eschborn, Germany), and subsequently blocked in PBS-Tween 20 containing 5% nonfat milk or 5% BSA. Membranes were then incubated overnight with primary antibodies directed against TMEM165 (Cat# HPA038299, 1:1000, Atlas Antibodies), decorin (Cat# MAB143, 1:1000, R&D Systems), HA (Cat# 901501, 1:10000, BioLegend), Myc (Cat# 2276, 1:1000, CST), Smad2 (Cat# 5339, 1:1000, CST), pSmad2 (Cat# 3104, 1:1000, CST), Smad1 (Cat# 6944, 1:1000, CST), pSmad1,5,9, (Cat# 13820, 1:1000, CST) CamKIIα (Cat# 50049, 1ː1000, CST), pCamKII α (Cat# sc-12886-R, 1ː1000, Santa Cruz Biotechnology) p44/42 MAPK (Cat# 4695, 1:1000, CST) phospho-p44/42 (Cat# 4370, 1:1000, CST), β-actin, (Cat# 3700, 1:1000, CST), asporin (Cat# ab58741, 1:1000, Abcam), TGFβR2 (Cat# GTX37527, 1:1000, GeneTex) or BMPR2 (Cat# GTX60415, 1:1000, GeneTex) followed by incubation with horseradish peroxidase-conjugated secondary antibodies (Cat# 7074, 1:2000, CST or Cat# 7076, 1:2000 CST). Antibodies were diluted in 5% BSA/0.01% tween 20 in PBS. The blots were then developed using Clarity Western ECL substrate (BIO-RAD, Hercules, CA) according to the instructions of the manufacturer.

### Data analysis and statistical procedures

Each experiment was repeated at least three times independently. Quantitative data were expressed as mean ± S.D. Statistical analysis was performed with an unpaired two-tailed Student’s *t*-test, and effects were considered statistically significant at *P < 0.05. One representative immunoblot of three independent experiments was shown in results.

## Supplementary information

checklist

## Data Availability

All data needed to evaluate the conclusions in the paper are present in the paper. Additional data related to this paper may be requested from the corresponding author.
